# Identifying and functionally characterizing tissue-specific and ubiquitously expressed human lncRNAs

**DOI:** 10.18632/oncotarget.6859

**Published:** 2016-01-09

**Authors:** Chunjie Jiang, Yongsheng Li, Zheng Zhao, Jianping Lu, Hong Chen, Na Ding, Guangjuan Wang, Juan Xu, Xia Li

**Affiliations:** ^1^ College of Bioinformatics Science and Technology, Harbin Medical University, Harbin, China

**Keywords:** ubiquitously expressed lncRNAs, tissue-specific lncRNAs, genomic structure, epigenetic regulation, functional prediction

## Abstract

Recent advances in transcriptome sequencing have made it possible to distinguish ubiquitously expressed long non-coding RNAs (UE lncRNAs) from tissue-specific lncRNAs (TS lncRNAs), thereby providing clues to their cellular functions. Here, we assembled and functionally characterized a consensus lncRNA transcriptome by curating hundreds of RNA-seq datasets across normal human tissues from 16 independent studies. In total, 1,184 UE and 2,583 TS lncRNAs were identified. These different lncRNA populations had several distinct features. Specifically, UE lncRNAs were associated with genomic compaction and highly conserved exons and promoter regions. We found that UE lncRNAs are regulated at the transcriptional level (with especially strong regulation of enhancers) and are associated with epigenetic modifications and post-transcriptional regulation. Based on these observations we propose a novel way to predict the functions of UE and TS lncRNAs through analysis of their genomic location and similarities in epigenetic modifications. Our characterization of UE and TS lncRNAs may provide a foundation for lncRNA genomics and the delineation of complex disease mechanisms.

## INTRODUCTION

Long non-coding RNAs (lncRNAs) are defined as transcripts longer than 200 nt without a known protein-coding function [1]. Over the past decade, large-scale, next-generation transcriptomic sequencing has led to the discovery of tens of thousands of novel lncRNA transcripts, making them challenging to catalogue and functionally characterize. While only a small number of lncRNAs have been well studied, it is thought that lncRNAs interact with DNA, RNA and proteins to serve both tissue specific and ubiquitous functions by regulating chromatin organization, transcription and post-transcriptional modifications [2–5], as well as splicing [6] and translation [6, 7].

A complete catalogue of lncRNAs would provide a basis for classifying uncharacterized members of this RNA species. Currently, lncRNA classification relies on the attributes originally used to detect them. As summarized by Laurent *et al.*, lncRNAs can be classified based on ten different features, including four major characteristics: genomic location and context, effects on DNA sequence, functions, and targeting mechanisms [8]. However, it is worth noting that the regulatory effectiveness of lncRNAs is dependent on their expression. Many lncRNAs show tissue-specific (TS) expression patterns, often restricted to a single cell line, providing important clues about their specific functions within cells. Most recent researches have entailed analysis of small cohorts of tissues to detect lncRNAs highly expressed in a given tissue or cell type. In order to fully characterize TS lncRNAs, it is necessary to integrate the findings from these smaller transcriptome sequencing datasets across many different tissues.

In addition to TS lncRNAs, there are also ubiquitously expressed lncRNAs (UE lncRNAs), serving universal housekeeping functions. For example, Washietl *et al.* [9] found that some lncRNAs (e.g. TUG1) are expressed in all examined tissue types. By analyzing the RNA-seq data of the Illumina Human Body Map Project, Derrien *et al.* [10] also found that, although patterns of lncRNAs are more tissue-specific than protein-coding genes, about 11% of lncRNAs are detected in every tissue tested. Ubiquitously expressed genes are required for the maintenance of basal cellular functions that are essential for the existence of a cell, regardless of their cell-specific role in the tissue or organism [11]. However, there has been no systematic identification and functional analysis of UE lncRNAs. Fortunately, advances in RNA-seq, integrated datasets may provide the opportunity to investigate these outstanding questions.

In this study, we integrated 16 independent, publically available RNA-seq datasets, including 206 samples across more than twenty different tissues. We focused on the lncRNA transcriptome in normal tissue samples, identifying novel UE lncRNAs and refining lists of TS lncRNAs. We next analyzed multiple features of these two lncRNA subsets, including gene structure composition, evolutionary conservation, regulatory features, and functional prediction. Finally, we established a method to predict the functions of UE and TS lncRNAs using their genomic location and similarities in epigenetic modifications. By uncovering the expansive landscape of TS and UE lncRNAs, we provide the scientific community with a powerful starting point to begin investigating their biological relevance.

## RESULTS

### The lncRNA transcriptome displays both tissue-specific and ubiquitously expressed features

**Figure 1:**
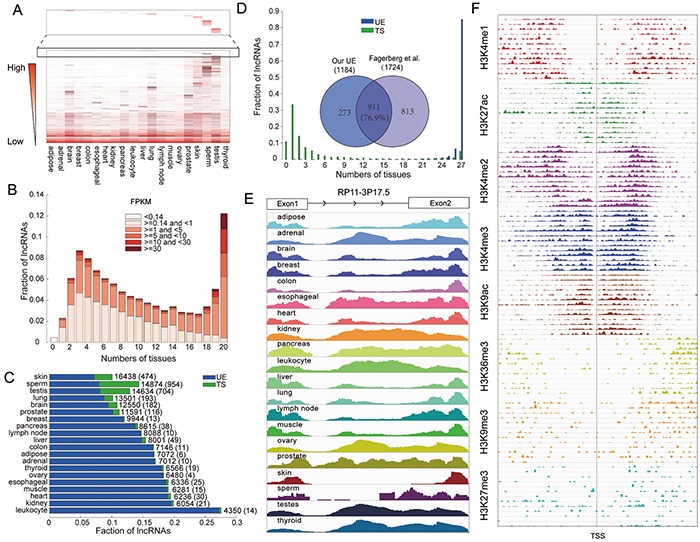
The lncRNA transcriptome exhibits both ubiquitously expressed and tissue-specific features **A.** Heat map of the whole lncRNA transcriptome. Dark color indicates higher expression, and light color indicates lower expression. **B.** LncRNA expression and number of tissues in which genes are expressed. **C.** Fraction of TS and UE lncRNAs in each tissue. The total numbers of expressed lncRNAs in each tissue are indicated. Values in brackets represent the number of lncRNAs that are tissue-specifically expressed in each tissue. **D.** The robustness of UE/TS lncRNAs is evaluated based on their expression width in an independent dataset. The Venn diagram illustrates the overlap between the UE lncRNA set identified in our integrated dataset and the UE lncRNA set identified in the independent analysis. **E.** An example UE lncRNA, RP11-3P17.5 (ENSG00000269888). **F.** The histone modifications of the RP11-3P17.5 promoter region in 13 cell lines, each line represents a different cell line.

We investigated the lncRNA transcriptome using publically available RNA-sequencing data from a diverse collection of human tissues (for details see methods). Based on the data from 94 normal samples across 20 tissue types (Supplementary Table S1), we found that 98.1% of lncRNAs and 88.5% of protein coding genes are detected using a fragments per kilobase of transcript per million mapped fragments (FPKM) threshold greater than 0. Thus, the integrated expression profile covers the majority of human lncRNAs and protein coding genes, suggesting that these data can be used to further investigate their expression patterns across different tissues. Similar to previous studies, we found that lncRNAs had lower expression than protein coding genes [10, 12] (Supplementary Figure S1A). After applying an FPKM threshold of 0.14 for lncRNAs and 0.21 for protein coding genes, which balanced the numbers of false positives and false negatives and controlled for expression differences (Supplementary Figure S1B and Supplementary Figure S1C, for details see methods), we found that the lncRNA transcriptome has both strong tissue-specific and ubiquitously expressed features (Figure 1A–1C). Using comparative analysis and calculating the expression width of lncRNAs, we revealed that, consistent with previous studies [10], a large proportion of lncRNAs show expression differences across different tissues. There are 2.3% of lncRNAs that are expressed in only one tissue, which is about 1.5 times more common than protein coding genes (Supplementary Figure S1D). In contrast, 12% of lncRNAs are expressed in all tissues (Figure 1A–1C and Supplementary Figure S1D). Interestingly, most lncRNAs expressed in all tissue types are highly expressed, whereas lncRNAs functioning in one tissue tend to have relatively low expression in the whole lncRNA transcriptome (Figure 1B). Thus, there is a positive correlation between lncRNA expression breadth and relative expression value (Figure 1B and Supplementary Figure S1E), suggesting that widely expressed lncRNAs may be the most important part of lncRNA transcriptome.

UE genes are required for basic cellular functions essential for cell viability. Thus, they are likely to be expressed in all cells of an organism under normal conditions, irrespective of tissue type. Based on the assumption that UE lncRNAs would also have universal expression across tissues, we identified 1,184 (6.4%) UE lncRNAs (Supplementary Table S2). We also identified 2,583 (14.0%) TS lncRNAs (Supplementary Table S2) which were expressed in only one tissue and had a high score of tissue specificity (as proposed by Cabili *et al.* [13]). To provide the convenient and available resource about the detailed information of UE/TS lncRNAs for biomedical scientists, Ubetis-LncDB, a free and web-accessible database, is further constructed (http://www.bio-bigdata.com/Ubetis-LncDB). In addition, we identified 5,619 (24.3%) UE and 2,824 (12.3%) TS protein coding genes following the same procedures. As shown in Figure 1C, the number of TS lncRNAs varies substantially across tissues, and has no correlation with the number of expressed lncRNAs in each tissue type. Consistent with previous studies [2, 14], the brain, testis, lung and skin tissues have more TS lncRNAs and TS protein coding genes, perhaps due to the presence of heterogeneous cell types in these tissues or from a need for more diverse lncRNA repertoires. Interestingly, the high number of TS lncRNA in testis has been discovered by several previous studies [9, 10, 13]. Both our study and Cabili *et al.* found that the testis tissue has the highest number of TS lncRNAs across the tissues considered, and 36.5% TS lincRNAs are also detected by Cabili *et al.*, which is significant. Thus, testis-specific lincRNAs may define a new class of RNAs in this organ. These results might be because this organ may represent a breeding ground for new genes, and may be due to the particularly efficient activity of proto-promoters in testis cells [9, 15]. Many TS lncRNAs are also found in other tissues. For example, two TS lncRNAs of the pancreas tissue, CTD-2503O16.4 (ENSG00000249856) and LINC00511 (ENSG00000227036) have been uncovered to be high-confidence human islet-cell genes [16]. On the contrary, the number of TS lncRNAs is lower in the breast, muscle and adipose tissues, reflecting more specialized functions of these tissues. In addition, we found that the TS lncRNAs overlap with those identified by Cabili *et al.* based on the K-means clustering with the tissue specificity distance measure (Supplementary Table S3).

In order to estimate the influence of different transcriptome datasets on prediction UE and TS lncRNAs, two additional datasets were analyzed (Supplementary Table S1). The first dataset was obtained from the Human Body Map 2 project [13] and had been included in the combined dataset analyzed above, and the second was an independent dataset assayed by Fagerberg *et al.* [17], containing 95 samples across 27 tissues. Consistent with our previous findings, we found that almost all UE lncRNAs are expressed in all tissues, 89% in Human Body Map 2 project and 86% in Fagerberg *et al.* respectively (Figure 1D and Supplementary Figure S1F). For TS lncRNAs, most are expressed in no more than two tissues. To further estimate the robustness of UE lncRNAs, we also identified UE lncRNAs in these two datasets based on the same criterion, and found a high degree of overlap (Figure 1D and Supplementary Figure S1F). The fraction of overlap is higher than the fraction of protein coding genes in previous studies which is about 50% [11, 17], indicating that UE lncRNAs exhibit higher robustness across different datasets. For example RP11-3P17.5 (ENSG00000269888), an intergenic lncRNA on chromosome 3q26.1 containing 2 exons, is expressed across all tissue types (Figure 1E). To further corroborate active transcription of this lncRNA, we intersected intervals surrounding the transcription start site (TSS) with ENCODE chromatin immunoprecipitation and sequencing (ChIP-seq) data. There were six activating and two repressive signals, suggesting that this lncRNA is actively transcribed (Figure 1F).

### UE lncRNAs have compact gene structure and high evolutionary conservation

**Figure 2:**
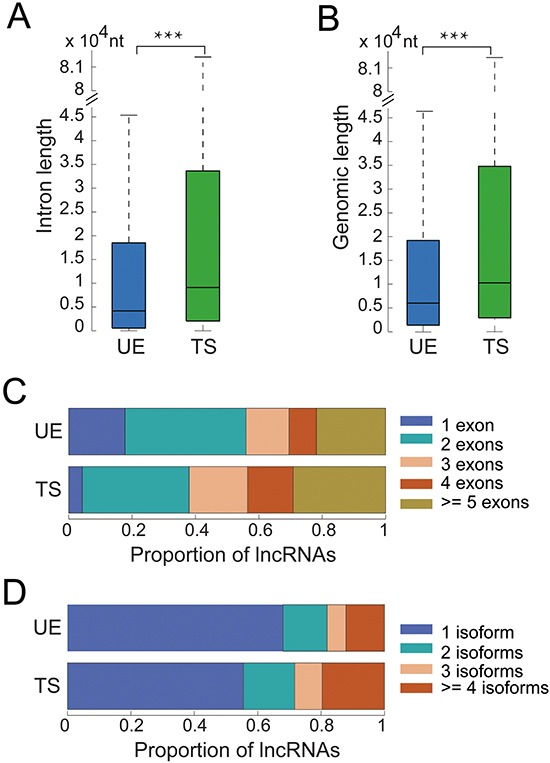
The genomic structure of UE/TS lncRNAs **A.** The total length of all introns for each UE/TS lncRNA gene. ***p < 6*10^−29^, Wilcoxon rank sum test. **B.** The total length of each UE/TS lncRNA gene containing both introns and exons. ***p < 5*10^−112^, Wilcoxon rank sum test. **C.** Number of exons for each UE/TS lncRNA gene. **D.** Number of transcripts for each UE/TS lncRNA gene.

We next characterized the genomic structure, evolutionary conservation, and transcriptional regulation of UE and TS lncRNAs. We observed that the medians of intronic and genomic lengths are 4,186 and 5,497 nt for UE lncRNAs, respectively versus 9,098 and 10,307 nt for TS genes (Figure 2A and 2B). Both measurements were shorter for UE lncRNAs than for TS lncRNAs (Wilcoxon rank sum test, *p* < 6*10^−29^ and *p* < 5*10^−112^, respectively). UE lncRNAs tended to have fewer exons and transcript isoforms than TS (Figure 2C and 2D). Over half of UE lncRNAs had only one or two exons, and over 80% of UE lncRNAs have only one or two transcripts. These observations indicate that UE lncRNAs have compact gene structure, consistent with the ‘selection for economy’ hypothesis which shows that natural selection appears to favor compact gene structure in highly expressed genes to minimize the cost of transcription and other molecular processes [18].

**Figure 3:**
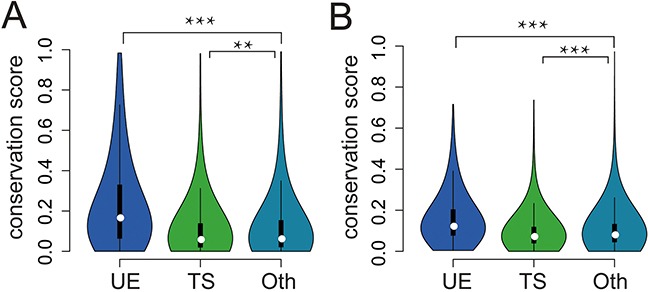
The conservation of UE/TS lncRNAs **A.** The conservation scores of exons for different lncRNA categories. Oth represents lncRNAs that are neither UE lncRNAs nor TS lncRNAs. Compared with Oth, UE lncRNAs are higher (*p* < 3*10^−102^, Wilcoxon rank sum test), while TS lncRNAs are lower (*p* =0.0048). **B.** The conservation scores of promoters for different lncRNA categories. Compared with Oth, UE lncRNAs are higher (*p* < 3.9*10^−80^, Wilcoxon rank sum test), while TS lncRNAs are lower (*p* < 3.8*10^−10^).

Human lncRNAs are under weaker selective constraints than protein coding genes [12, 19]; however, few attempts have been made to examine how UE lncRNAs evolve and how different they are from TS lncRNAs. Using pre-calculated, nucleotide-level calculations of evolutionary selection from the PhastCons algorithm [20], we found that UE lncRNAs had the highest conservation (Figure 3A). In addition, it was reported that lncRNA promoters are almost as conserved as protein coding gene promoters [10, 19]. Here, we also found the highest conservation for UE lncRNA promoters (Figure 3B). In summary, both UE lncRNA exons and promoters are under the strongest purifying selection pressures among the lncRNA transcriptome, indicating the important roles of UE lncRNAs.

### Expression of UE lncRNAs is tightly regulated

**Figure 4:**
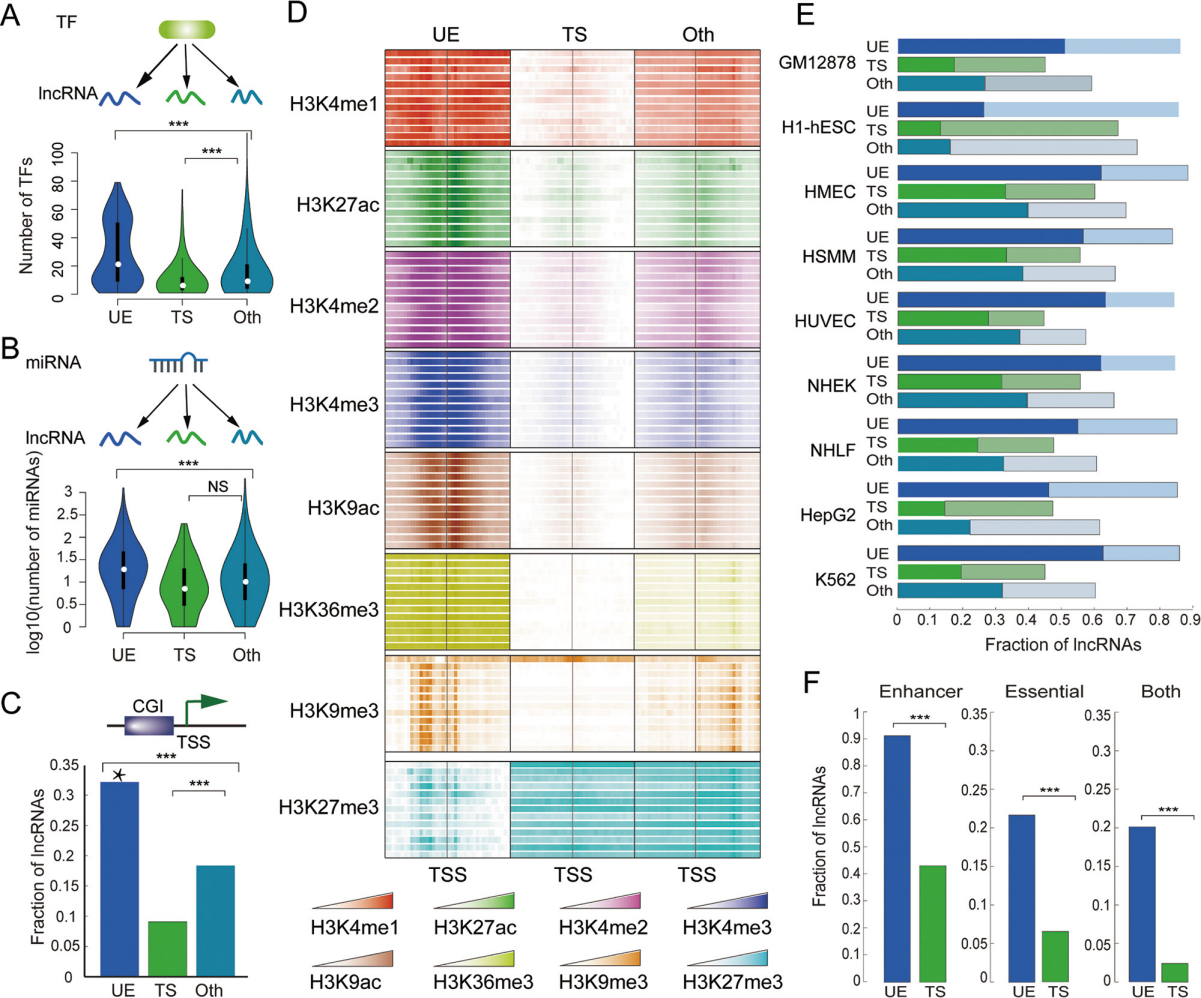
UE lncRNAs are strictly regulated by TF, miRNAs, and epigenetic modification The distribution of TFs **A.** and miRNAs **B.** that target each lncRNA. ***p < 2*10^−7^, NS (nonsense) *p* =0.073, Wilcoxon rank sum test. **C.** The distribution of CpG islands. The Y-axis represents the fraction of lncRNAs whose promoter contains at least one CpG island. ***p < 1*10^−27^, Fisher's exact test. Star above the bar means significant enrichment (hypergeometric test, *p* < 1*10^−32^) of CpG islands. **D.** Histone modification signals in lncRNA promoters across 13 cell lines. **E.** Distribution of strong and weak enhancers. The X-axis represents the fraction of lncRNAs whose 10KB up- and downstream region overlap with enhancers in each examined cell line. The dark bars represent strong enhancers, and the light bars represent weak enhancers. **F.** Distribution of UE/TS enhancers and essential genes. Left: the fraction of UE/TS lncRNAs whose 10KB up- and downstream region overlap with only UE/TS enhancers. Middle: the fraction of UE/TS lncRNAs whose 10KB up- and downstream region overlap with at least one essential gene. Right: the fraction of UE/TS lncRNAs whose 10KB up- and downstream region overlap with at least one essential gene and only UE/TS enhancers. ***p < 1*10^−37^, Fisher's exact test.

Expression of lncRNAs is tightly regulation both at the transcriptional and post-transcriptional levels [2, 21, 22]. However, how ubiquitous or tissue-specific expression is achieved is not clear. To illuminate these questions, we calculated the number of transcription factors (TFs) and miRNAs targeting each lncRNA, and found that UE lncRNAs are under the strictest regulation (both transcriptional and post-transcriptional); whereas TS lncRNAs are regulated by the lowest number of TFs and miRNAs (Figure 4A and 4B). Proteins with central roles in signaling pathways or protein-protein interaction networks tend to be strictly regulated by TFs and miRNAs [21, 23], thus UE lncRNAs might also serve important functions and need to respond to a wide variety of signals in order to perform their functions.

Similar to protein coding genes, most lncRNAs are transcribed by RNA pol II., and Pol II-mediated gene expression is regulated by DNA methylation and histone modifications [24]. CpG-islands (CGIs) are found at the promoters of most UE protein coding genes [25]. Similarly, we found that about 1/3 of UE lncRNA promoters have CGIs, indicating enrichment (Figure 4C, hypergeometric test, *p* < 1*10^−32^). In contrast, the promoters of TS lncRNAs do not often have CGIs, and TS lncRNAs also have poor GC content (Figure 4C and Supplementary Figure S2A).

On the other hand, recent deep-sequencing technologies have made it possible to examine the histone modification patterns at genome-wide level and thus enable a more concrete description of different kinds of lncRNAs [26, 27]. Here, we investigated histone modification patterns at lncRNA promoters, and found that UE lncRNAs frequently exhibit six types of activating modifications: H3K4me1, H3K27ac, H3K4me2, H3K4me3, H3K9ac, H3K36me3, but few repressive signals (such as H3K27me3) across thirteen different cell types obtained from ENCODE (Figure 4D). These results indicate that the combination of both high active modifications and low repressive signals might contribute to the high and universal expression of UE lncRNAs. Interestingly, there is a distinct pattern of high-density H3K9me3 marks across TSSs of UE lncRNAs, whereas there are few modifications near the TSSs of TS lncRNAs.

Chromatin marks also correspond with other genomic elements, such as enhancers, which are marked by H3K4me1 and H3K27ac in a wide range of cell types. Enhancers are short DNA regions and assume strong, weak, and poised states that correlate with neighboring gene expression and function [28]. Using the identified enhancers by the chromHMM model [29], we found that UE lncRNAs are more likely to be regulated by adjacent enhancers under different distance thresholds (Figure 4E and Supplementary Figure S2B–S2C). Key promoter sequence elements are differentially distributed between genes with different functions, including elements that are predominantly found at either developmentally regulated or at UE genes [30]. Moreover, following the definition proposed by Zabidi *et al*. [30], enhancers are further classified into UE enhancers and TS enhancers, where UE enhancers are active in at least two cell types, while developmental enhancers exhibit strong cell-type specificity. Using these criteria, we found that the vast majority of UE lncRNAs are near only UE enhancers, and TS lncRNAs are near only enhancers with TS activity (Figure 4F and Supplementary Figure S2D–S2E).

lncRNAs are closely linked with development, and enhancers are also enriched for lncRNAs near developmental and cell type-specific genes, reinforcing their roles as sentinels of precise gene expression. To explore the co-localization of UE lncRNAs and essential protein coding genes, we looked for genes that are the human orthologs of mouse genes which, when disrupted by homologous recombination, result in embryonic or postnatal lethality (Mouse Genome Informatics; www.informatics.jax.org). We then calculated the genomic distance between these essential genes and UE or TS lncRNAs. Within a distance of 50kb, there is at least one essential gene around 34.5% of UE lncRNAs; however, the proportion for TS lncRNAs is only 12.1% (Figure 4F and Supplementary Figure S2D–S2E). Moreover, for TS lncRNAs with only a TS enhancer, this proportion is reduced to 2.59%. Therefore, we conclude that a large number of UE lncRNAs are also essential for human development or survival.

### Functions of UE lncRNAs can be predicted based on neighboring protein coding genes

**Figure 5:**
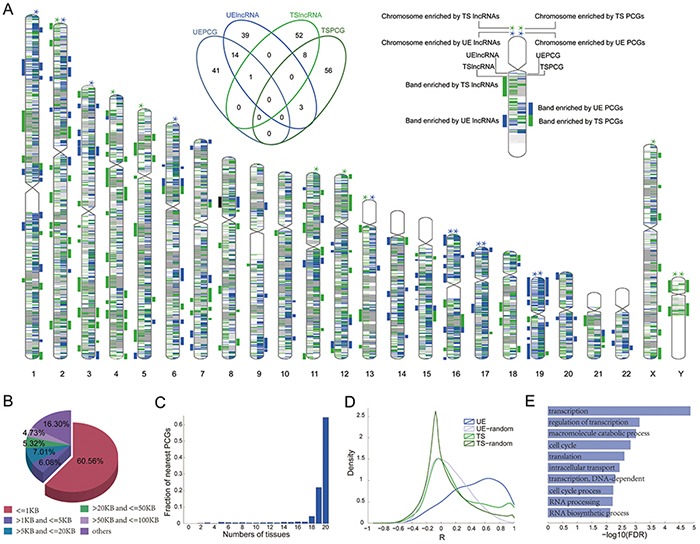
Functions of UE lncRNAs can be predicted based on their neighboring protein coding genes **A.** Distribution of UE/TS lncRNAs in the whole chromosome and chromosome bands. The stars above chromosomes denote enrichment by UE or TS genes (Hypergeometric test, *p* < =0.05). Bars to the side of chromosomes denote enrichment in a band (green bar= enriched by UE genes; blue bar= enriched by TS genes; black bar= enriched by both UE and TS genes). The Venn diagram illustrates the overlap among enriched chromosome bands by different gene categories. **B.** Fraction of UE lncRNAs adjacent to UE protein coding genes within different distances. **C.** The expression width of the most proximal protein coding genes for UE lncRNAs. **D.** The co-expression distribution between UE/TS lncRNAs and their neighbor protein coding genes. The lines marked UE (or TS) represent the co-expression distribution between UE (or TS) lncRNAs and their neighbor protein coding genes. UE-random (or TS-random) represents the random co-expression corresponding to UE (or TS) lncRNAs, respectively. **E.** Enriched GO terms for the protein coding genes within the 5KB distance of UE lncRNA genes.

Currently, there are two commonly used methods to predict lncRNA function: 1) based on their co-expression with protein coding genes, or 2) genomic co-localization with protein coding genes [31–33]. We investigated the genomic distributions of lncRNAs and found that both UE lncRNAs and TS lncRNAs are dispersed throughout multiple chromosomes; however, several chromosomes are enriched (Figure 5A, chromosome layout). Notably, TS lncRNAs are specifically enriched on chromosome Y, and 95% of these TS lncRNAs are specifically expressed in the testis and sperm tissues, while none of the UE lncRNAs are located in chromosome Y. Few UE and TS lncRNAs are not found within the same chromosome bands (Figure 5A, Venn diagram and chromosome layout); however, there is co-localization of UE lncRNAs and UE protein coding genes, with 15 of 57 enriched chromosome bands of UE lncRNAs overlapping and 10 adjacent to those bands enriched by UE protein coding genes.

Next, we investigated whether UE lncRNAs tend to be neighbors with UE protein coding genes. Surprisingly, 83.7% of UE lncRNAs are adjacent to UE protein coding genes within 100KB up- and downstream of UE lncRNAs (Figure 5B). Even focusing on the nearest neighbors, there are still about 60% of UE lncRNAs surrounded by UE protein coding genes. Moreover, 90.64% of proximal protein coding genes are expressed in at least 90% of tissues we analyzed (Figure 5C), and the expression of UE lncRNAs is positively correlated with the expression of neighboring UE protein coding genes (Figure 5D). Hence, we believe that the function of UE lncRNAs could be predicted based on their neighboring protein coding genes, when both co-localization and co-expression are considered. Indeed, after performing functional enrichment analysis for UE lncRNAs, we found enrichment for basic cell maintenance (Figure 5E, Supplementary Table S4).

### Integrating expression and epigenetic similarities to predict the TS lncRNA function

**Figure 6:**
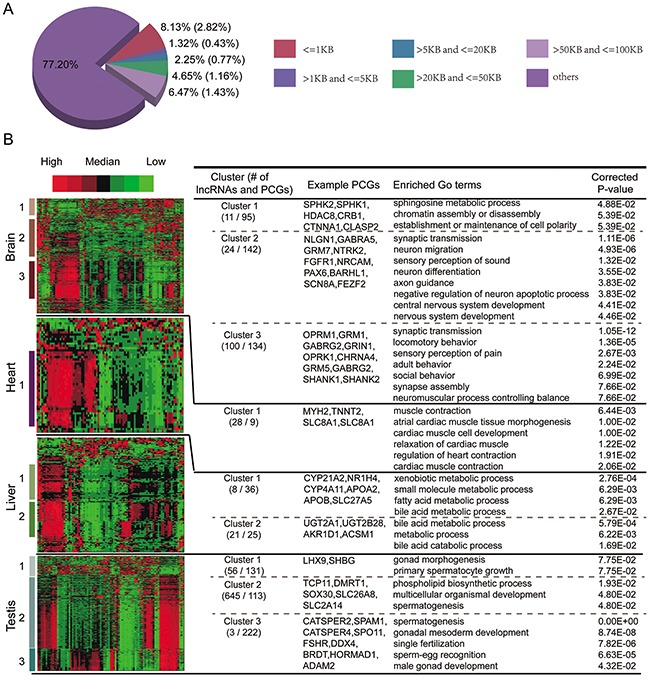
Functional predictions of TS lncRNAs **A.** Fraction of TS lncRNAs adjacent to TS protein coding genes within different distances. The values in brackets represent the fraction of TS lncRNAs that are tissue-specifically expressed in the same tissue as their neighbor TS protein coding genes. **B.** Hierarchical clustering of the active histone modification profiles of both TS lncRNAs and TS protein coding genes. Enriched GO terms and example TS protein coding genes are shown to the right.

For TS lncRNAs, although there are several chromosome bands that are also overrepresented by TS protein coding genes, this overlap is not significant (Figure 5A). Moreover, only about 23% of TS lncRNAs have TS protein coding genes within 100KB up- or downstream (Figure 6A). When we required that the TS lncRNAs and their corresponding nearest neighbor TS protein coding genes must be specifically expressed in the same tissues, the proportion was reduced to 6.6%. In addition, we found that TS lncRNAs and their neighboring protein coding genes do not tend to be co-expressed (Figure 5D). Thus, unlike UE lncRNAs, the neighboring protein coding genes of TS lncRNAs do not tend to be tissue-specifically expressed, let alone in the same tissues.

An alternative way to predict the function of TS lncRNAs is based on TS protein coding genes expressed in the same tissue. Despite similar expression patterns, groups of functionally related protein coding genes or lncRNAs can be distinguished at the level of chromatin [26, 34]. Thus, we proposed an integrative framework to predict the function of TS lncRNAs by considering both co-expression and co-modification. All six types of active signals were selected and, for each tissue, we performed clustering analysis of epigenetic modifications to simultaneously group both TS lncRNAs and TS protein coding genes. The function of TS lncRNAs could be predicted based on the TS protein coding genes within the same clusters (see methods). As a result, several dominant clusters and corresponding biological processes were identified (Figure 6B). For example, several liver-specific expressed lncRNAs are enriched in liver-related functions, such as bile acid metabolic, bile acid and bile salt transport, lipid transport, cholesterol catabolic process, etc.

## DISCUSSION

UE genes are universally expressed in all tissue and cell types and constitute the basal transcriptome for the maintenance of basic cellular functions. Identification of UE genes facilitates exploration of the underlying cellular infrastructure and increases understanding of structural genomic features. In this study, we systematically identified 1,184 UE lncRNAs based on an integrated lncRNA transcriptome. As examples, both TUG1 and SNHG6 were ubiquitously expressed in our study, and they are also expressed in all human tissues in the lncrnadb database [35]. These two lncRNAs regulate basal cellular functions [19, 36–38]. TUG1, an intergenic lncRNA, is involved in multiple development processes and diseases [39]. Moreover, we uncovered a range of features that are specific to UE lncRNAs, including compact gene structure, high conservation, strict combinational regulation at transcriptional, post-transcriptional, and epigenetic levels, and strong regulation of enhancers. Our systematic analysis of UE lncRNAs will provide a missing link between function and expression of UE lncRNAs. In addition, UE lncRNAs tend to be genomically co-localized and co-expressed with UE protein coding genes. As a consequence, it is possible to predict the functions of UE lncRNAs using these common methods.

Most researchers currently focus their attention on TS lncRNAs, and we were able to confirm many TS lncRNAs based on our integrated dataset. Consistent with earlier reports, the brain and testis express most TS lncRNAs [2, 14]. Several known brain-specific lncRNAs were also confirmed in our study, including MIAT and RNCR3. Previous studies found that RNCR3 is conserved and exhibits dynamic expression in retinal development [40, 41]. Another example is PCGEM1, which is prostate tissue-specific and prostate cancer-associated [42], and whose overexpression promotes cell proliferation [43]. Interestingly, TS lncRNAs do not tend to colocalize with TS protein coding genes, and are not often co-expressed with neighboring genes, making it unreasonable to predict TS lncRNA function using common methods. Therefore, we proposed an integrative framework to predict TS lncRNA functions by combining co-expression with epigenetic similarities.

When studying lncRNAs, it is straightforward to investigate their functional features by classifying them into different groups. Currently, the existing classifications of lncRNAs rest on their descriptive and distinctive properties: from their size, to their localization, to their function [8, 44, 45]. For example, the GENCODE database also classifies lncRNAs into lincRNA or antisense RNA, in addition to intron-associated biotypes [46]. However, classification of lncRNAs is highly dependent on the current existing knowledge, thus requiring frequent validation of the classification system, exploring new classification systems. Acted as one kind of regulatory RNA molecules, identifying both UE and TS lncRNAs would be necessary to make easier interpretation of lncRNA functionality. Indeed, we discovered their several distinct features as well as functions. Moreover, the different classes are not mutually exclusive. For example, TUG1, an intergenic lncRNA, is a UE lncRNA and has been found to regulate the basal cellular functions, which are important in multiple development processes and diseases. We also found that 76.5% TS lncRNA are located at the intergenic regions. Thus, we believe that the classification of both UE and TS lncRNAs is of fundamental importance for lncRNA studies, helpful for further analysis of specific lncRNAs, for formulation of new hypothesis based on expression features of lncRNAs and for exploration of the underlying lncRNA functional mechanisms.

Another interesting use for TS lncRNAs is for identification of candidate markers and pharmacologic targets [47, 48], which are also differentially expressed in disease. Here, we identified potential TS lncRNA markers in four datasets based on edgeR [49] (Supplementary Table S5). Globally, these differentially expressed TS lncRNAs can distinguish disease samples from normal samples (Supplementary Figure S3). Many of these TS lncRNA markers are novel, but some have been previously reported. For example, lncRNA MIR17HG (ENSG00000215417) is specifically expressed in lung, and its expression is down regulated in non-small cell lung cancer. Inhibited expression of miR-18a, transcribed from MIR17HG, increases tumor growth and lung metastasis [14].

When studying lncRNAs, it is straightforward to predict their functions by classifying them into different groups. Currently, the existing classifications of lncRNAs rely upon several properties: size, localization, and function [8, 44, 45]. For example, the GENCODE database further classifies lncRNAs into lincRNA or antisense RNA in addition to intron-associated biotypes [47]. However, classification of lncRNAs is highly dependent on existing knowledge, which means identifying UE vs. TS lncRNAs is necessary for interpretation of their functionality. Indeed, we discovered features and functions that distinguish UE and TS lncRNAs, but also found that there is overlap in these two groups, particularly in intergenic regions. We believe that the classification of UE and TS lncRNAs is of fundamental importance for further analysis of specific lncRNAs, for formulation of new hypotheses based on lncRNA features, and for exploration of lncRNA functions.

## MATERIALS AND METHODS

### Gene annotation

A comprehensive set of lncRNA annotation was integrated from three resources, including Ensembl (GRCH37), GENCODE v18, and Cabili *et al.* [13]. To construct a non-redundant lncRNA set, we compared the localization of lncRNAs from each dataset. If the overlap of two lncRNA loci was larger than 0.8, only the lncRNA in GENCODE was reserved. Ultimately, there were 18,404 lncRNAs examined. Protein coding gene annotations were also from Ensembl, and 23,087 protein coding genes were analyzed.

### Integrated RNA-seq expression datasets

We searched the GEO database [50] to obtain the transcriptome data from available normal tissues based on the following steps: first, RNA-seq data was collected using three key words (RNA-seq, tissue, and human); then, the samples were kept only if (i) they were normal tissues of adult, (ii) from extracted total RNA, and (iii) the platform used was illumina. In total, 94 samples belonging to 20 tissues were collected, and the transcriptome from the Human Body Map 2 were also included [13]. Another dataset from the Human Protein Atlas was used as an independent dataset to assess the robustness of UE/TS lncRNAs identified in our study, which included 95 human individuals representing 27 different tissues [17]. In addition, to analyze the expression changes in diseased tissues, we also obtained 17 disease samples corresponding to 16 of the 94 normal samples. The detailed information for these samples is listed in Supplementary Table S1.

The sequence reads were mapped to the human genome (hg19) using Tophat [51]. To obtain quantification scores for all human transcripts of both lncRNAs and protein coding genes, FPKM (fragments per kilobase of exon per million fragments mapped) values were calculated using Cufflinks v2.1.1 [52]. In addition, for tissues with multiple samples, the highest expression value of a gene among samples of the tissue was selected to represent its expression in the tissue.

### Conservation information

PhastCons scores [20] for 46 vertebrate genomes were downloaded from UCSC. The conservation of both exon and the promoter regions were analyzed. The regions 2 kb up- and downstream of transcription start sites were defined as promoters. The average PhastCons score at each nt position of each region was computed to analyze conservation.

### Regulation by TFs and miRNAs

The transcriptional regulation of lncRNAs was extracted from ChIPBase [53], a database for decoding the transcriptional regulation from ChIP-seq data. At a distance within 5 kb upstream and 1kb downstream of each lncRNA TSS, there were 132,996 TF-lncRNA regulatory relationships, including 120 TF binding sites and 9,022 lncRNAs interactions. In addition, CLIP-supported sites between miRNA and lncRNA were identified by integrating the available AGO-CLIP peak clusters from starBase V2.0 [54] and the predicted sites of Miranda (August 2010 release) [55]. The default parameters of Miranda were used to identify miRNA target sites in full-length lncRNA transcripts. Ultimately, 38,776 miRNA-lncRNA regulatory relationships including 1,085 lncRNAs were obtained.

### Epigenetic regulation data

Both DNA methylation and histone modifications were analyzed. The degree of DNA methylation was measured based on CGI and GC content. The coordinates of CGIs were downloaded from UCSC, and categorization of promoters by CpG content was performed as described in [56]. In addition, eight histone modifications in 13 human cell lines were analyzed, which were assayed by Chip-seq and obtained from the ENCODE project [57]. To investigate the distinct histone modification pattern on lncRNA promoters, the promoter region of each lncRNA was divided into equal 40 bins (100 nt for each bin). The average number of reads in each bin of all lncRNAs belonging to a specified gene category was calculated. Previously identified enhancers were also obtained from published sources [29, 58]. According to a previous study [30], enhancers were further classified as UE or TS enhancers based on the number of detected tissues. If an enhancer was found in more than two cell lines, it was defined as a UE enhancer; otherwise, it was labeled a TS enhancer.

### Identification of UE and TS lncRNAs

A transcript was defined as a UE transcript only if it was expressed above a certain cutoff FPKM value in all tissues examined and if the coefficient of variance of its FPKM value across tissues was less than 1. A transcript was defined as a TS transcript if it was expressed in just one tissue and its tissue specificity score was above 0.4, which was calculated based on the method proposed by Cabili *et al.* [13]. After that, we defined a lncRNA gene as UE or TS only if it expressed at least one transcript meeting the UE/TS criteria.

For the expression threshold of expression, it was not reasonable to choose the same value for lncRNAs and protein coding genes, because lncRNAs are much more lowly expressed than protein coding genes. In order to increase the accuracy of our results, we computed different thresholds based on different backgrounds for lncRNAs and protein coding genes. Based on a previous study [59], a comparison between the expression of transcriptional regions and un-transcribed regions was used to find a threshold for detectable expression above background (Supplementary Figure S1A and Supplementary Figure S1B), yielding threshold FPKM values of 0.14 for lncRNAs and 0.21 for protein coding genes, which balanced the numbers of false positives and false negatives and considered the relative expression of lncRNAs and protein coding genes.

### Functional enrichment analysis

After detecting the associated gene sets for UE/TS lncRNAs, the hypergeometric test was used for finding enriched Gene Ontology (GO) categories. Then, the enriched significance *P* values were adjusted by Benjamini and Hochberg methods and finally, GO terms with adjusted *P* values < 0.1 and including at least two interesting genes were considered.

### Randomization tests

To test whether UE (or TS) lncRNAs were co-expressed with their neighbor protein coding genes, we calculated their correlation coefficients. We then randomly selected the same number of protein coding genes from the background set as their pseudo-neighbors and recomputed the paired correlation coefficients. This procedure was repeated 1,000 times.

### Statistical analysis

The Wilcoxon rank sum test was used to assess the differences among gene categories in genomic length, conservation and the number of TFs and miRNAs that target the genes. The enrichment of CpG islands and lncRNA sets in different chromosomes and chromosome bands were assessed by hypergeometric tests. The distributions of CpG islands, enhancers and essential genes among different lncRNA categories were assessed by Fisher's exact test. Differential gene expression was identified using edgeR [49].

## SUPPLEMENTARY FIGURES AND TABLES










