# Supplementary material for: Identifying and functionally characterizing tissue-specific and ubiquitously expressed human lncRNAs
Source: Oncotarget. 2016 Jan 9;7(6):7120–33. doi: 10.18632/oncotarget.6859 (PMC4872773; doi:10.18632/oncotarget.6859)
Supplement: Supplementary file 1 [file oncotarget-07-7120-s001.pdf]

SUPPLEMENTARY FIGURES AND TABLES

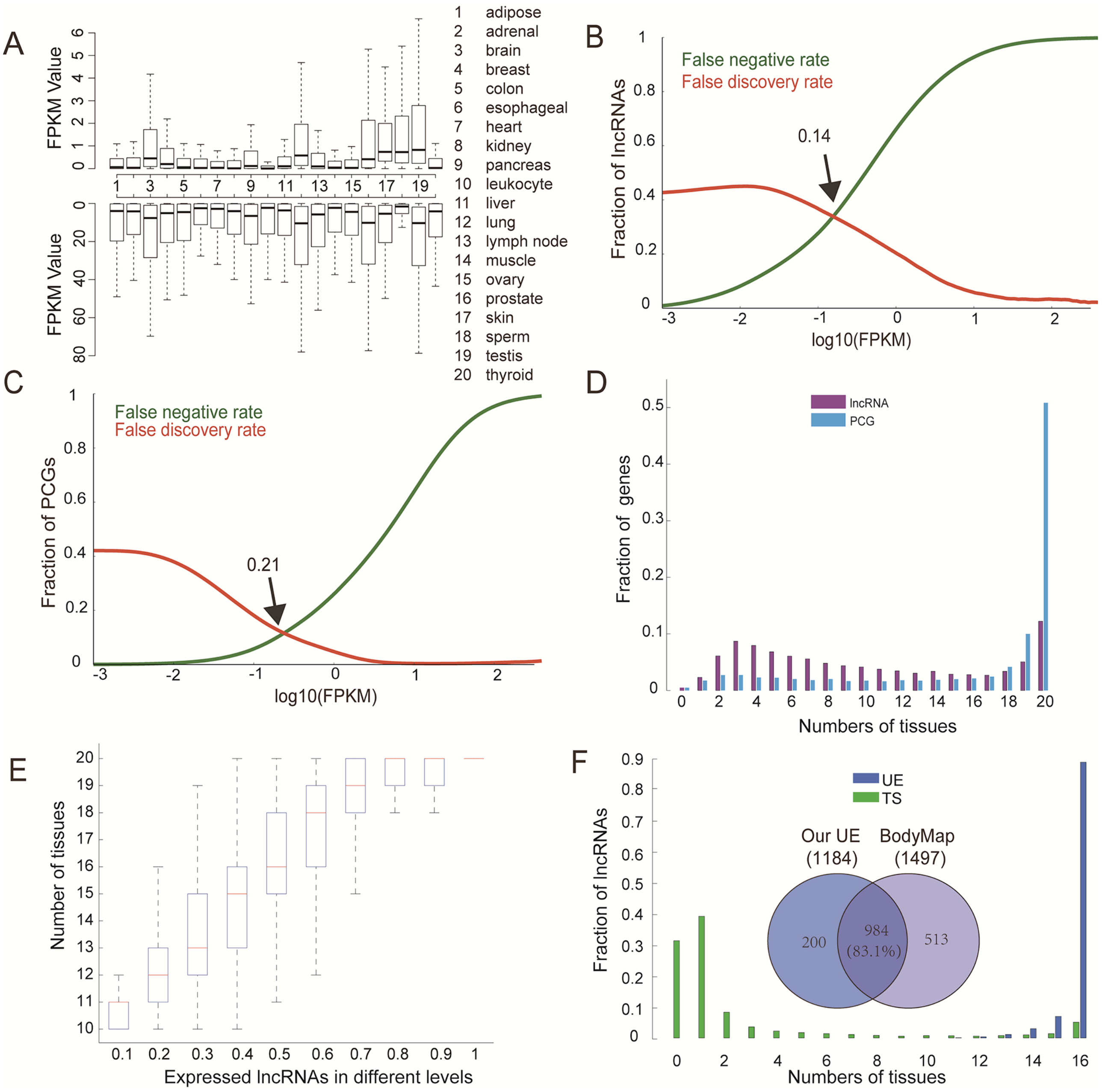

Supplementary Figure S1: The expression pattern of lncRNAs. A. The expression value (FPKM) of both lncRNAs (Upper) and protein coding genes (Lower) in each tissue. Each bar represents a tissue. **B.** False discovery and negative rate for the detection of genes as a function of detection threshold used, demonstrating how a threshold of 0.14 FPKM for lncRNAs was chosen. **C.** A threshold of 0.21 FPKM for protein coding genes (PCGs) was chosen. **D.** Distribution of the number of tissues in which lncRNAs and protein coding genes (PCGs) are expressed (FPKM >=0.14 for lncRNA and FPKM >=0.21 for protein coding gene). **E.** The correlation between expression breadth and expression levels. The median expression value of a lncRNA in 20 tissues was chosen as the expression of the lncRNA. LncRNAs were then ranked in descending order, and were divided into ten equal parts. **F.** The part of distribution graph shows the distribution of the number of tissues in which UE/TS lncRNAs are expressed in Human Body Map 2 dataset. The Venn diagram illustrates the comparison between UE lncRNA set identified in our integrated dataset and the UE lncRNA set identified in datasets of Human Body Map 2.

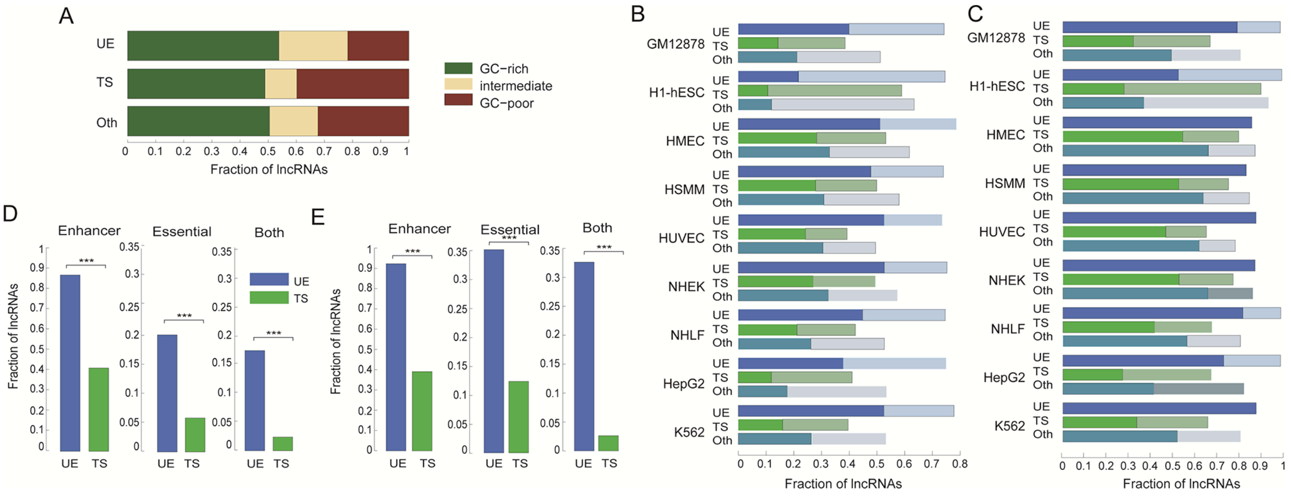

Supplementary Figure S2: UE lncRNAs are strictly regulated. A. The distribution of GC-content in different lncRNA categories. **B.** Similar with figure 4E, but for 5KB up- and downstream region of each lncRNA. **C.** Similar with figure 4E, but for 50KB up- and downstream region of each lncRNA. **D.** Similar with figure 4F, but for 5KB up- and downstream region of each lncRNA. **E.** Similar with figure 4F, but for 50KB up- and downstream region of each lncRNA.

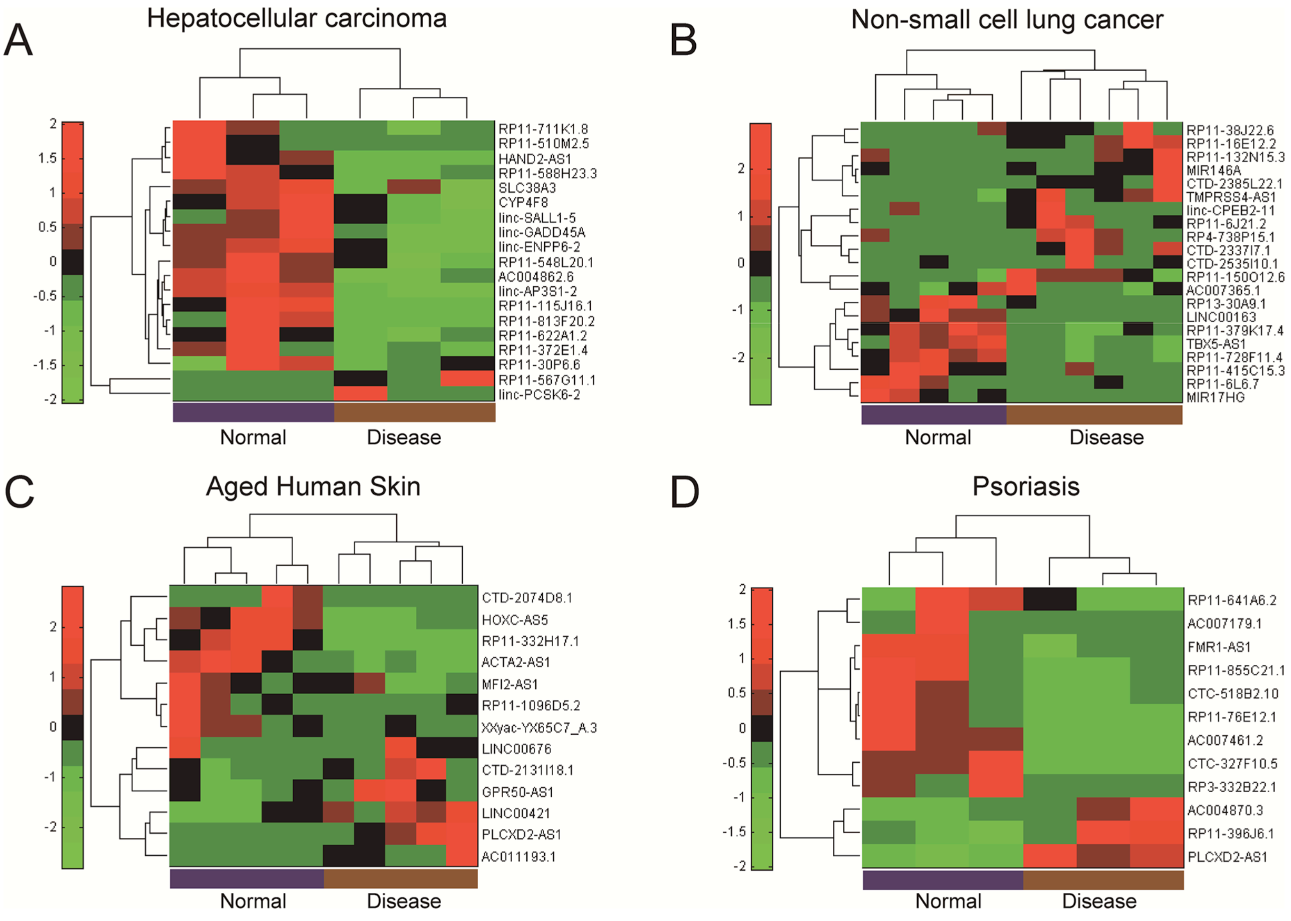

Supplementary Figure S3: Differentially expressed TS lncRNAs can separate disease samples and normal samples. Heatmaps show disease samples can be distinguished from normal based on the expression of differentially expressed TS lncRNAs. (Columns) Samples. (Rows) LncRNAs. In the color scheme, red indicates higher expression, and green indicates lower expression. Four datasets: hepatocellular carcinoma **A.** non-small cell lung cancer **B.** aged human skin **C.** and psoriasis **D.**

Supplementary Table S1: RNA-seq datasets used in this study.
See Supplementary File 1

Supplementary Table S2: The detail information of UE/TS lncRNAs.
See Supplementary File 2

Supplementary Table S3: Comparison of TS lncRNAs between us and Cabili *et al*.

Tissue	OurLnc	OurLinc	Ratio	P
adipose_breast	19	14	0.142857	0.003019
adrenal_lymphNode	21	15	0.266667	1.72E-05
brain	182	121	0.206612	0
colon	11	5	0.6	9.45E-07
heart	30	25	0.52	0
kidney	21	15	0.533333	1.29E-13
liver	49	36	0.416667	0
lung	193	131	0.045802	0.000208
muscle	15	9	0.111111	0.037512
prostate	116	74	0.027027	0.079383
testes	704	580	0.365517	0
thyroid	19	14	0.285714	5.41E-06

The common tissues shared by us and Cabili *et al*. are showed in the first column. The second column represents the number of TS lncRNAs. Considering that lncRNAs from Cabili *et al*. are lincRNAs, the corresponding number of TS lincRNAs is showed. The ratio represent the fraction of TS lncRNAs which are also identified in same tissue by Cabili *et al* based on the K-means clustering with the tissue specificity distance measure. And the *p* values coming from hypergeometric distribution represent the significance of the overlap between our TS lncRNAs and those identified by Cabili *et al*.

Supplementary Table S4:Enriched GO terms for the neighour protein coding genes of UE lncRNAs under different distance thresholds.
See Supplementary File 3

Supplementary Table S5: The list of differentially expressed TS lncRNAs.
See Supplementary File 4
